# 10-(4-Chloro­phen­yl)-4-[(4-fluoro­phen­yl)amino]-5-phenyl-5,8,9,10-tetra­hydro­pyrimido[4,5-*b*]quin­olin-6(7*H*)-one

**DOI:** 10.1107/S2414314625007758

**Published:** 2025-09-05

**Authors:** Adesola A. Adeleke, Sizwe J. Zamisa, Bernard Omondi

**Affiliations:** a School of Agriculture and Science, Discipline of Chemistry, University of KwaZulu-Natal, Private Bag X54001, Durban, 4000, South Africa; Katholieke Universiteit Leuven, Belgium

**Keywords:** crystal structure, pyrimidine, *syn*-clinal orientation

## Abstract

The title compound, C_29_H_22_ClFN_4_O, crystallizes with one mol­ecule in the asymmetric unit. The pyrimidinyl and anilinyl moieties subtend a dihedral angle = 36.65 (5)°, while the di­hydro­pyridine and phenyl rings are approximately orthogonal [dihedral angles = 81.65 (7) and 89.95 (7)°]. The crystal packing reveals alternating C—H⋯π and C—F⋯π inter­actions forming corrugated two-dimensional sheets along the *ac* plane, which are further linked into a three-dimensional network through N—H⋯O and C—H⋯O hydrogen bonds and Cl⋯Cl inter­actions.

## Abstract 2

The crystal structure of the title compound features a single asymmetric unit with distinct *syn*-clinal and orthogonal ring arrangements, forming corrugated two-dimensional sheets *via* C—H⋯π and C—F⋯π inter­actions that are further reinforced into a three-dimensional network by C—H⋯O, N—H⋯O hydrogen bonds and Cl⋯Cl contacts.

## Graphical abstract

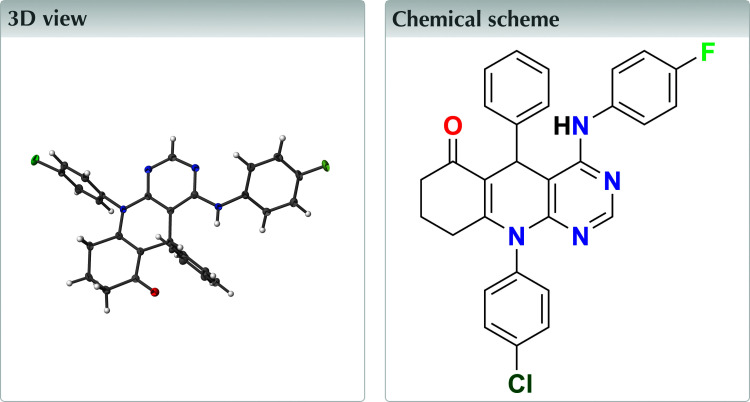

## Structure description

The title compound represents a heterocycle from the pyrimido[4,5-*b*]quinoline family, distinguished by its fused pyrimidine and quinoline framework. These scaffolds are typically constructed *via* efficient single-pot, multicomponent reactions (Moosavi-Zare & Najafi, 2023 ▸). Fused tetra­hydro­quinoline systems, especially those incorporating pyrimidine units, continue to attract pharmaceutical research due to their broad-spectrum bioactivity – including anti­microbial, anti­cancer, anti­malarial, anti-inflammatory, and anti­histaminic potential (Patel *et al.*, 2024 ▸; Tawfeek *et al.*, 2024 ▸). Beyond these applications, pyrimidine motifs have shown utility in modulating signaling enzymes such as Abl kinase and PTP1B, and can also function as DNA inter­calators (Esmaili *et al.*, 2022 ▸). Motivated by ongoing efforts to develop enhanced pyrimidine-quinoline-based therapeutics, we now report the crystallographic characterization of the title compound (Zamisa *et al.*, 2023 ▸).

**Figure 1:**
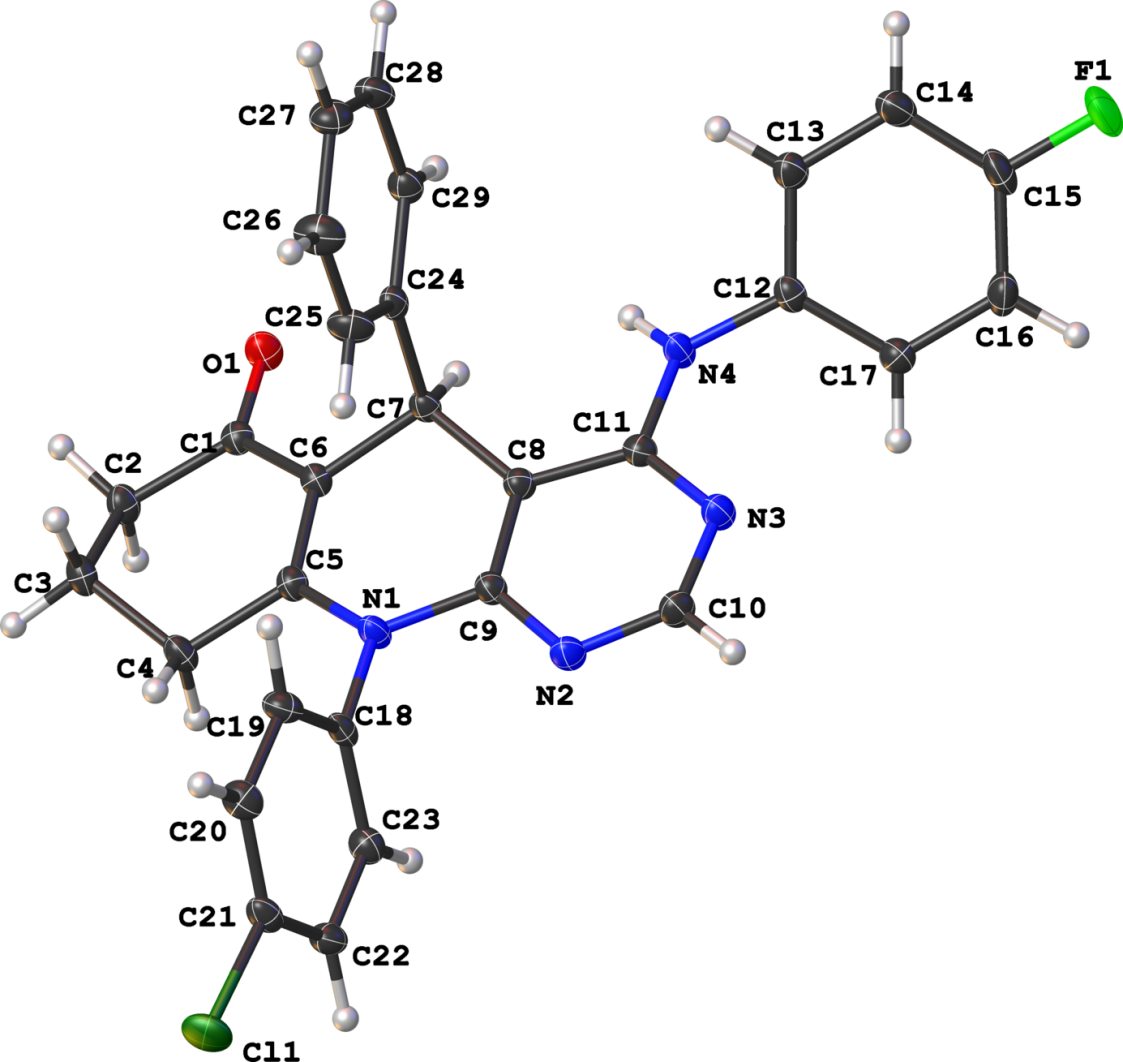
Mol­ecular structure of the title compound with displacement ellipsoids drawn at the 50% probablility level.

The crystal structure of the title compound consists of one mol­ecule in the asymmetric unit (Fig. 1 ▸). The pyrimidinyl and anilinyl units exhibit near coplanarity, with a dihedral angle of 36.65 (7)°. In contrast, the di­hydro­pyridine and the C–C18 and C24–C29 phenyl rings are nearly perpendicular, subtending dihedral angles of 81.65 (7) and 89.85 (7)°, respectively. These geometric parameters are comparable with those of reported for chromeno­pyrimidine (Zamisa *et al.* 2022 ▸) and hexa­hydro­quinolinyl formimidate (Zamisa & Omondi, 2022 ▸) derivatives

**Figure 2:**
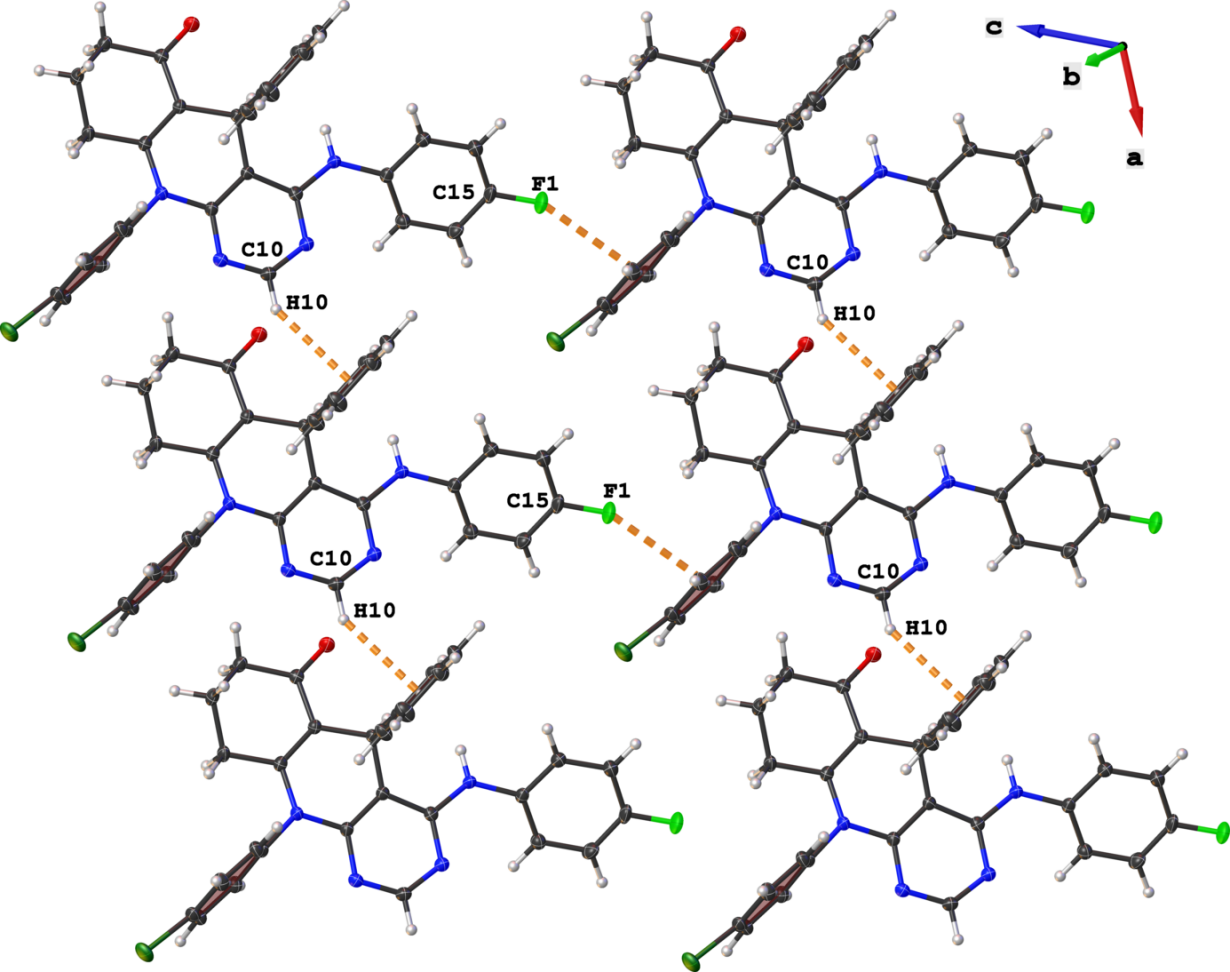
Representation of the C10—H10⋯*Cg*1 and C15—F1⋯*Cg*2 inter­actions in the crystal packing of the title compound.

**Figure 3:**
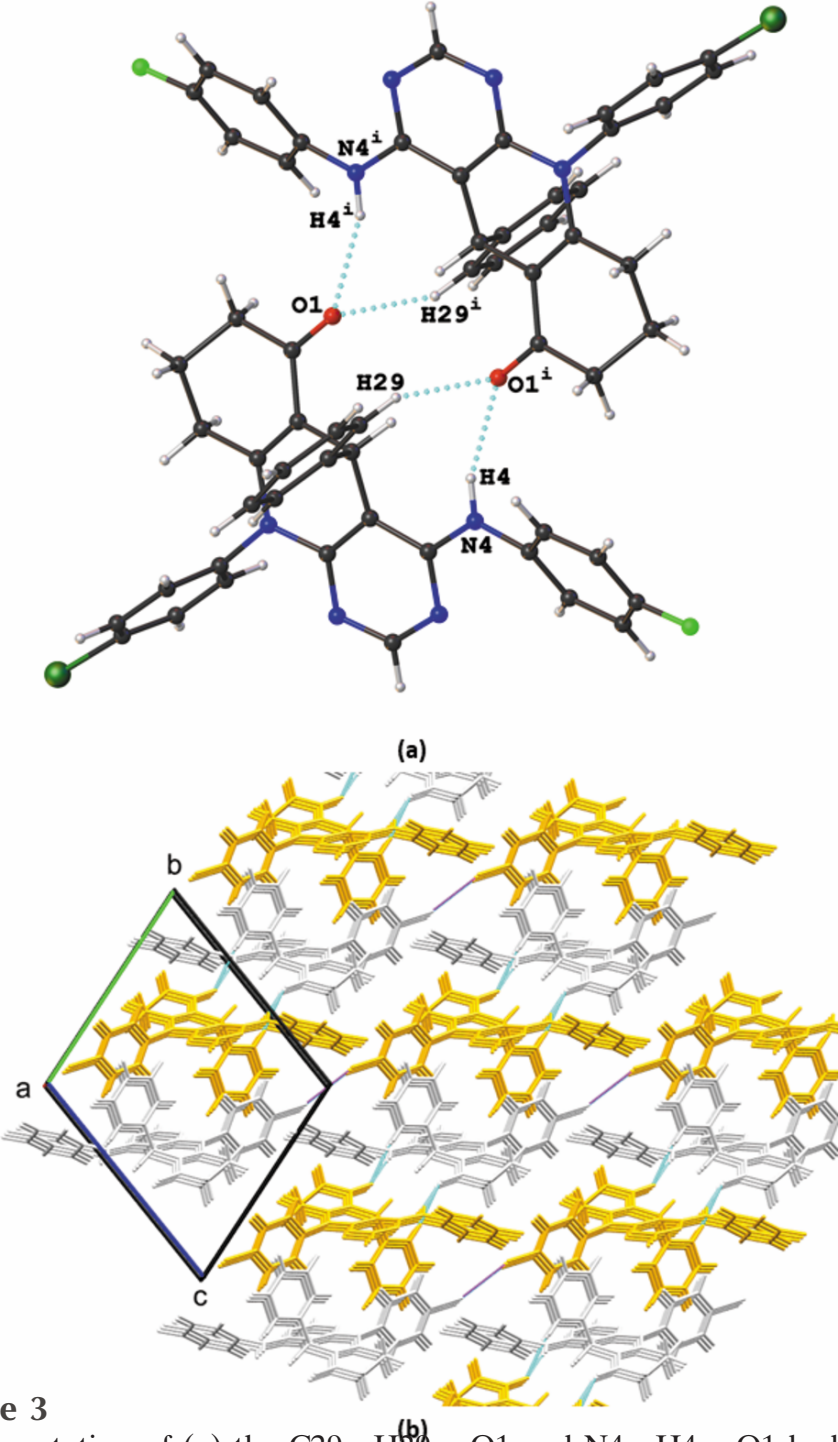
Representation of (*a*) the C29—H29⋯O1 and N4—H4⋯O1 hydrogen bonds in the crystal packing of the title compound and (*b*) the formation of a three dimensional supramolecular structure formed *via* alternating double-acceptor hydrogen bonds involving the O1 atom (cyan-coloured dashed lines) and Cl⋯Cl contacts (magenta-coloured dashed lines). Gold and grey colors indicate symmetry relationships with the asymmetric unit. Light grey represents the asymmetric unit contents, while golden-yellow indicates inversion symmetry. Symmetry code: (i) −*x*, −*y*, −*z* + 1.

In the packing of the title compound, C—H⋯π hydrogen bonds are observed between the H10 atom of the pyrimidine ring and the centroid of the C24–C20 phenyl ring (*Cg*1), which form supra­molecular chains along the crystallographic *a-*axis direction (Table 1 ▸). These chains are linked *via* C15—F1⋯*Cg*2 inter­actions (*Cg*2 is the centroid of the C18–C23 ring) to form supra­molecular sheets that extend alongthe [111] direction (Fig. 2 ▸, Table 1 ▸). The O1 atom of the carbonyl group acts as a double acceptor for the N4—H4⋯O1 and C29—H29⋯O1 hydrogen bonding patterns, which together with Cl1⋯Cl1 contacts [3.2755 (5) Å < 3.50 Å ; (sum of van der Waals radii), symmetry code: −*x* + 2, −*y* + 2, −*z* + 2], link the sheets into a three-dimensional supra­molecular architecture (Fig. 3 ▸, Table 1 ▸).

## Synthesis and crystallization

The stepwise inter­mediates: 2-amino-1-(4-chloro­phen­yl)-5-oxo-4-phenyl­hexa­hydro­quinoline-3-carbo­nitrile and ethyl (*E*)-*N*-(3-cyano-1-(4-chloro­phen­yl)-5-oxo-4-phenyl­hexa­hydro­quinolin-2-yl)formimidate were synthesized *via* adapted literature protocols (Zamisa *et al.*, 2022 ▸; Zamisa & Omondi, 2022 ▸). Following established methods, the synthesis involved combining 1 mmol of the formimidate derivative with 1.2 mmol of 4-fluoro­aniline in 10 ml of acetic acid. This mixture was sealed in a 30 ml high-pressure vial and subjected to microwave irradiation (200 W) at 413 K for 20 minutes using a single-mode microwave reactor. The formation of the product was confirmed using thin-layer chromatography (TLC). After the reaction, distilled water was carefully layered onto the mixture, creating a cloudy suspension that was left undisturbed overnight. The resulting solid was harvested by vacuum filtration, rinsed with distilled water, and purified *via* recrystallization using an ethanol–water solvent system (Zamisa *et al.*, 2023 ▸).

## Refinement

Crystal data, data collection and structure refinement details are summarized in Table 2 ▸.

## Supplementary Material

Crystal structure: contains datablock(s) I. DOI: 10.1107/S2414314625007758/vm4071sup1.cif

Structure factors: contains datablock(s) I. DOI: 10.1107/S2414314625007758/vm4071Isup2.hkl

Supporting information file. DOI: 10.1107/S2414314625007758/vm4071Isup3.cml

CCDC reference: 2484308

Additional supporting information:  crystallographic information; 3D view; checkCIF report

## Figures and Tables

**Table 1:** Hydrogen-bond geometry (Å, °) *Cg*1 and *Cg*2 are the centroids of the C24–C29 and C18–C23 rings, respectively.

*D*—H⋯*A*	*D*—H	H⋯*A*	*D*⋯*A*	*D*—H⋯*A*
N4—H4⋯O1^i^	0.88	2.17	3.0036 (16)	159
C29—H29⋯O1^i^	0.95	2.46	3.3169 (18)	150
C10—H10⋯*Cg*1^ii^	0.95	2.74	3.5719 (18)	147
C15—F1⋯*Cg*2^iii^	1.3604 (18)	3.1604 (13)	4.4286 (18)	154.70 (10)

Symmetry codes: (i) 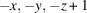; (ii) 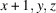; (iii) 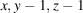.

**Table 2:** Experimental details

Crystal data	
Chemical formula	C_29_H_22_ClFN_4_O
*M*_r_	496.95
Crystal system, space group	Triclinic, *P* 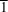
Temperature (K)	100
*a*, *b*, *c* (Å)	8.4512 (2), 11.6756 (3), 12.7892 (3)
α, β, γ (°)	106.100 (1), 101.492 (2), 97.612 (2)
*V* (Å^3^)	1164.29 (5)
*Z*	2
Radiation type	Mo *K*α
μ (mm^−1^)	0.20
Crystal size (mm)	0.23 × 0.14 × 0.08

Data collection	
Diffractometer	Bruker SMART APEX2 area detector
Absorption correction	Multi-scan (*SADABS*; Krause et al., 2015 ▸)
*T*_min_, *T*_max_	0.696, 0.746
No. of measured, independent and observed [*I* > 2σ(*I*)] reflections	17352, 5250, 4225
*R*_int_	0.027
(sin θ/λ)_max_ (Å^−1^)	0.651

Refinement	
*R*[*F*^2^ > 2σ(*F*^2^)], *wR*(*F*^2^), *S*	0.038, 0.095, 1.04
No. of reflections	5250
No. of parameters	325
H-atom treatment	H-atom parameters constrained
Δρ_max_, Δρ_min_ (e Å^−3^)	0.30, −0.26

Computer programs: *COSMO* and *SAINT* (Bruker, 2009 ▸), *SHELXT2018/2* (Sheldrick, 2015*a* ▸), *SHELXL2018/3* (Sheldrick, 2015*b* ▸) and *OLEX2* (Dolomanov *et al.*, 2009 ▸).

## full crystallographic data

### 10-(4-Chlorophenyl)-4-[(4-fluorophenyl)amino]-5-phenyl-5,8,9,10-tetrahydropyrimido[4,5-b]quinolin-6(7H)-one Crystal data

**Table:**

C_29_H_22_ClFN_4_O	*Z* = 2
*M**_r_* = 496.95	*F*(000) = 516
Triclinic, *P*1	*D*_x_ = 1.418 Mg m^−^^3^
*a* = 8.4512 (2) Å	Mo *K*α radiation, λ = 0.71073 Å
*b* = 11.6756 (3) Å	Cell parameters from 5292 reflections
*c* = 12.7892 (3) Å	θ = 2.5–27.5°
α = 106.100 (1)°	µ = 0.20 mm^−^^1^
β = 101.492 (2)°	*T* = 100 K
γ = 97.612 (2)°	Block, colourless
*V* = 1164.29 (5) Å^3^	0.23 × 0.14 × 0.08 mm

### 10-(4-Chlorophenyl)-4-[(4-fluorophenyl)amino]-5-phenyl-5,8,9,10-tetrahydropyrimido[4,5-b]quinolin-6(7H)-one Data collection

**Table:**

Bruker SMART APEX2 area detector diffractometer	5250 independent reflections
Radiation source: microfocus sealed X-ray tube, Incoatec Iµs	4225 reflections with *I* > 2σ(*I*)
Mirror optics monochromator	*R*_int_ = 0.027
Detector resolution: 7.9 pixels mm^-1^	θ_max_ = 27.6°, θ_min_ = 1.7°
ω and φ scans	*h* = −10→9
Absorption correction: multi-scan (SADABS; Krause et al., 2015)	*k* = −15→15
*T*_min_ = 0.696, *T*_max_ = 0.746	*l* = −16→15
17352 measured reflections	

### 10-(4-Chlorophenyl)-4-[(4-fluorophenyl)amino]-5-phenyl-5,8,9,10-tetrahydropyrimido[4,5-b]quinolin-6(7H)-one Refinement

**Table:**

Refinement on *F*^2^	Primary atom site location: dual
Least-squares matrix: full	Hydrogen site location: inferred from neighbouring sites
*R*[*F*^2^ > 2σ(*F*^2^)] = 0.038	H-atom parameters constrained
*wR*(*F*^2^) = 0.095	*w* = 1/[σ^2^(*F*_o_^2^) + (0.0402*P*)^2^ + 0.494*P*] where *P* = (*F*_o_^2^ + 2*F*_c_^2^)/3
*S* = 1.03	(Δ/σ)_max_ < 0.001
5250 reflections	Δρ_max_ = 0.30 e Å^−^^3^
325 parameters	Δρ_min_ = −0.26 e Å^−^^3^
0 restraints	

### 10-(4-Chlorophenyl)-4-[(4-fluorophenyl)amino]-5-phenyl-5,8,9,10-tetrahydropyrimido[4,5-b]quinolin-6(7H)-one Special details

**Table:**

Geometry. All esds (except the esd in the dihedral angle between two l.s. planes) are estimated using the full covariance matrix. The cell esds are taken into account individually in the estimation of esds in distances, angles and torsion angles; correlations between esds in cell parameters are only used when they are defined by crystal symmetry. An approximate (isotropic) treatment of cell esds is used for estimating esds involving l.s. planes.

### 10-(4-Chlorophenyl)-4-[(4-fluorophenyl)amino]-5-phenyl-5,8,9,10-tetrahydropyrimido[4,5-b]quinolin-6(7H)-one Fractional atomic coordinates and isotropic or equivalent isotropic displacement parameters (Å^2^)

**Table:**

	*x*	*y*	*z*	*U*_iso_*/*U*_eq_	
Cl1	0.86634 (5)	0.87170 (3)	0.96121 (3)	0.02694 (11)	
F1	0.45178 (13)	−0.26576 (10)	−0.02464 (9)	0.0397 (3)	
O1	−0.04527 (13)	0.11981 (9)	0.62009 (9)	0.0196 (2)	
N1	0.45496 (15)	0.39153 (11)	0.68315 (10)	0.0170 (3)	
N2	0.65678 (15)	0.34370 (11)	0.58815 (11)	0.0184 (3)	
N3	0.60496 (15)	0.16517 (11)	0.42959 (11)	0.0186 (3)	
N4	0.35389 (15)	0.02975 (11)	0.36641 (10)	0.0175 (3)	
H4	0.260621	0.004335	0.381264	0.021*	
C1	0.05053 (18)	0.21608 (12)	0.67702 (12)	0.0150 (3)	
C2	0.02320 (19)	0.28912 (13)	0.78701 (13)	0.0188 (3)	
H2A	0.066013	0.253542	0.846130	0.023*	
H2B	−0.096591	0.283690	0.779822	0.023*	
C3	0.10726 (19)	0.42222 (13)	0.82264 (13)	0.0189 (3)	
H3A	0.051144	0.462085	0.771092	0.023*	
H3B	0.098103	0.463853	0.899330	0.023*	
C4	0.28759 (19)	0.43447 (13)	0.82097 (12)	0.0184 (3)	
H4A	0.335833	0.520915	0.834679	0.022*	
H4B	0.347684	0.408114	0.882085	0.022*	
C5	0.30843 (18)	0.35904 (13)	0.71031 (12)	0.0153 (3)	
C6	0.19369 (18)	0.25977 (12)	0.64127 (12)	0.0145 (3)	
C7	0.21029 (17)	0.18858 (12)	0.52702 (12)	0.0143 (3)	
H7	0.170754	0.100014	0.514119	0.017*	
C8	0.38970 (18)	0.20947 (13)	0.52368 (12)	0.0147 (3)	
C9	0.50129 (18)	0.31190 (13)	0.59558 (12)	0.0155 (3)	
C10	0.69670 (19)	0.26784 (13)	0.50356 (13)	0.0194 (3)	
H10	0.804490	0.289713	0.494794	0.023*	
C11	0.45157 (18)	0.13464 (13)	0.44097 (12)	0.0157 (3)	
C12	0.38757 (18)	−0.04232 (12)	0.26750 (12)	0.0158 (3)	
C13	0.25837 (19)	−0.08672 (13)	0.17169 (13)	0.0197 (3)	
H13	0.154546	−0.064739	0.174021	0.024*	
C14	0.2785 (2)	−0.16261 (14)	0.07267 (14)	0.0242 (4)	
H14	0.189555	−0.194182	0.007472	0.029*	
C15	0.4307 (2)	−0.19067 (14)	0.07178 (14)	0.0246 (4)	
C16	0.5628 (2)	−0.14599 (14)	0.16411 (14)	0.0228 (3)	
H16	0.667351	−0.165706	0.159818	0.027*	
C17	0.54137 (19)	−0.07179 (13)	0.26349 (13)	0.0192 (3)	
H17	0.630809	−0.041216	0.328430	0.023*	
C18	0.56396 (18)	0.50712 (13)	0.74583 (12)	0.0158 (3)	
C19	0.53198 (19)	0.61016 (14)	0.71943 (13)	0.0201 (3)	
H19	0.444946	0.603477	0.656895	0.024*	
C20	0.6271 (2)	0.72343 (13)	0.78427 (13)	0.0205 (3)	
H20	0.605018	0.794967	0.767541	0.025*	
C21	0.75403 (19)	0.73003 (13)	0.87341 (13)	0.0184 (3)	
C22	0.79132 (19)	0.62711 (14)	0.89766 (13)	0.0194 (3)	
H22	0.882328	0.633451	0.957559	0.023*	
C23	0.69449 (19)	0.51452 (13)	0.83365 (13)	0.0188 (3)	
H23	0.717561	0.442984	0.849934	0.023*	
C24	0.10579 (18)	0.22623 (13)	0.43494 (12)	0.0150 (3)	
C25	0.1398 (2)	0.34558 (14)	0.43307 (13)	0.0220 (3)	
H25	0.229200	0.402228	0.488211	0.026*	
C26	0.0455 (2)	0.38264 (14)	0.35238 (14)	0.0261 (4)	
H26	0.070133	0.464417	0.352350	0.031*	
C27	−0.0857 (2)	0.30057 (14)	0.27089 (13)	0.0223 (3)	
H27	−0.151043	0.326085	0.215382	0.027*	
C28	−0.12015 (19)	0.18154 (14)	0.27138 (13)	0.0212 (3)	
H28	−0.209032	0.124906	0.215720	0.025*	
C29	−0.02512 (18)	0.14469 (13)	0.35305 (13)	0.0181 (3)	
H29	−0.049798	0.062880	0.352987	0.022*	

### 10-(4-Chlorophenyl)-4-[(4-fluorophenyl)amino]-5-phenyl-5,8,9,10-tetrahydropyrimido[4,5-b]quinolin-6(7H)-one Atomic displacement parameters (Å^2^)

**Table:**

	*U*^11^	*U*^22^	*U*^33^	*U*^12^	*U*^13^	*U*^23^
Cl1	0.0285 (2)	0.01610 (18)	0.0258 (2)	−0.00600 (15)	−0.00005 (16)	−0.00021 (15)
F1	0.0370 (6)	0.0433 (6)	0.0267 (6)	0.0056 (5)	0.0150 (5)	−0.0121 (5)
O1	0.0196 (5)	0.0164 (5)	0.0212 (6)	−0.0007 (4)	0.0071 (4)	0.0039 (4)
N1	0.0161 (6)	0.0147 (6)	0.0156 (6)	−0.0013 (5)	0.0047 (5)	−0.0009 (5)
N2	0.0154 (6)	0.0177 (6)	0.0186 (7)	−0.0005 (5)	0.0041 (5)	0.0018 (5)
N3	0.0162 (6)	0.0189 (6)	0.0184 (7)	0.0009 (5)	0.0061 (5)	0.0021 (5)
N4	0.0147 (6)	0.0170 (6)	0.0176 (7)	−0.0012 (5)	0.0071 (5)	0.0003 (5)
C1	0.0163 (7)	0.0144 (7)	0.0157 (7)	0.0043 (6)	0.0040 (6)	0.0061 (6)
C2	0.0206 (8)	0.0184 (7)	0.0187 (8)	0.0034 (6)	0.0095 (6)	0.0050 (6)
C3	0.0237 (8)	0.0161 (7)	0.0180 (8)	0.0056 (6)	0.0097 (6)	0.0032 (6)
C4	0.0214 (8)	0.0159 (7)	0.0153 (8)	0.0011 (6)	0.0065 (6)	0.0005 (6)
C5	0.0178 (7)	0.0148 (7)	0.0147 (7)	0.0042 (6)	0.0057 (6)	0.0050 (6)
C6	0.0165 (7)	0.0136 (7)	0.0140 (7)	0.0032 (5)	0.0047 (6)	0.0043 (6)
C7	0.0145 (7)	0.0119 (6)	0.0142 (7)	−0.0001 (5)	0.0038 (6)	0.0017 (5)
C8	0.0147 (7)	0.0157 (7)	0.0135 (7)	0.0021 (5)	0.0041 (6)	0.0041 (6)
C9	0.0163 (7)	0.0157 (7)	0.0140 (7)	0.0029 (6)	0.0045 (6)	0.0036 (6)
C10	0.0156 (7)	0.0206 (7)	0.0204 (8)	0.0010 (6)	0.0061 (6)	0.0042 (6)
C11	0.0161 (7)	0.0156 (7)	0.0147 (7)	0.0027 (6)	0.0029 (6)	0.0044 (6)
C12	0.0187 (7)	0.0124 (6)	0.0161 (7)	0.0010 (5)	0.0068 (6)	0.0031 (6)
C13	0.0178 (8)	0.0206 (7)	0.0193 (8)	0.0022 (6)	0.0063 (6)	0.0033 (6)
C14	0.0220 (8)	0.0258 (8)	0.0185 (8)	−0.0002 (7)	0.0043 (6)	0.0000 (7)
C15	0.0305 (9)	0.0217 (8)	0.0190 (8)	0.0036 (7)	0.0128 (7)	−0.0016 (6)
C16	0.0210 (8)	0.0203 (7)	0.0275 (9)	0.0077 (6)	0.0103 (7)	0.0034 (7)
C17	0.0194 (8)	0.0180 (7)	0.0183 (8)	0.0037 (6)	0.0039 (6)	0.0034 (6)
C18	0.0169 (7)	0.0140 (7)	0.0137 (7)	−0.0009 (6)	0.0068 (6)	−0.0005 (6)
C19	0.0203 (8)	0.0218 (8)	0.0145 (8)	0.0010 (6)	0.0012 (6)	0.0033 (6)
C20	0.0254 (8)	0.0160 (7)	0.0199 (8)	0.0017 (6)	0.0055 (6)	0.0062 (6)
C21	0.0189 (8)	0.0149 (7)	0.0173 (8)	−0.0028 (6)	0.0058 (6)	0.0003 (6)
C22	0.0177 (8)	0.0221 (8)	0.0147 (8)	0.0018 (6)	0.0018 (6)	0.0026 (6)
C23	0.0215 (8)	0.0170 (7)	0.0177 (8)	0.0041 (6)	0.0051 (6)	0.0048 (6)
C24	0.0143 (7)	0.0168 (7)	0.0142 (7)	0.0027 (5)	0.0069 (6)	0.0032 (6)
C25	0.0221 (8)	0.0189 (7)	0.0200 (8)	−0.0036 (6)	−0.0001 (6)	0.0053 (6)
C26	0.0310 (9)	0.0190 (8)	0.0261 (9)	−0.0001 (7)	0.0012 (7)	0.0100 (7)
C27	0.0228 (8)	0.0261 (8)	0.0176 (8)	0.0046 (7)	0.0016 (6)	0.0085 (7)
C28	0.0182 (8)	0.0223 (8)	0.0169 (8)	−0.0008 (6)	0.0017 (6)	0.0008 (6)
C29	0.0184 (7)	0.0147 (7)	0.0190 (8)	0.0013 (6)	0.0048 (6)	0.0027 (6)

### 10-(4-Chlorophenyl)-4-[(4-fluorophenyl)amino]-5-phenyl-5,8,9,10-tetrahydropyrimido[4,5-b]quinolin-6(7H)-one Geometric parameters (Å, º)

**Table:**

Cl1—C21	1.7398 (15)	C12—C13	1.388 (2)
F1—C15	1.3604 (18)	C12—C17	1.395 (2)
O1—C1	1.2325 (17)	C13—H13	0.9500
N1—C5	1.3850 (19)	C13—C14	1.386 (2)
N1—C9	1.4020 (18)	C14—H14	0.9500
N1—C18	1.4453 (17)	C14—C15	1.370 (2)
N2—C9	1.3467 (19)	C15—C16	1.376 (2)
N2—C10	1.3274 (19)	C16—H16	0.9500
N3—C10	1.3287 (19)	C16—C17	1.386 (2)
N3—C11	1.3454 (19)	C17—H17	0.9500
N4—H4	0.8800	C18—C19	1.382 (2)
N4—C11	1.3643 (18)	C18—C23	1.383 (2)
N4—C12	1.4163 (19)	C19—H19	0.9500
C1—C2	1.509 (2)	C19—C20	1.388 (2)
C1—C6	1.456 (2)	C20—H20	0.9500
C2—H2A	0.9900	C20—C21	1.378 (2)
C2—H2B	0.9900	C21—C22	1.381 (2)
C2—C3	1.521 (2)	C22—H22	0.9500
C3—H3A	0.9900	C22—C23	1.385 (2)
C3—H3B	0.9900	C23—H23	0.9500
C3—C4	1.517 (2)	C24—C25	1.393 (2)
C4—H4A	0.9900	C24—C29	1.390 (2)
C4—H4B	0.9900	C25—H25	0.9500
C4—C5	1.503 (2)	C25—C26	1.378 (2)
C5—C6	1.363 (2)	C26—H26	0.9500
C6—C7	1.512 (2)	C26—C27	1.392 (2)
C7—H7	1.0000	C27—H27	0.9500
C7—C8	1.515 (2)	C27—C28	1.384 (2)
C7—C24	1.528 (2)	C28—H28	0.9500
C8—C9	1.3823 (19)	C28—C29	1.388 (2)
C8—C11	1.409 (2)	C29—H29	0.9500
C10—H10	0.9500		
			
C5—N1—C9	120.50 (12)	C13—C12—C17	119.61 (14)
C5—N1—C18	120.58 (12)	C17—C12—N4	123.14 (13)
C9—N1—C18	118.91 (12)	C12—C13—H13	119.5
C10—N2—C9	114.19 (12)	C14—C13—C12	121.03 (15)
C10—N3—C11	116.06 (13)	C14—C13—H13	119.5
C11—N4—H4	116.7	C13—C14—H14	121.0
C11—N4—C12	126.52 (12)	C15—C14—C13	117.91 (15)
C12—N4—H4	116.7	C15—C14—H14	121.0
O1—C1—C2	120.18 (13)	F1—C15—C14	118.51 (15)
O1—C1—C6	120.95 (13)	F1—C15—C16	118.71 (15)
C6—C1—C2	118.84 (12)	C14—C15—C16	122.79 (15)
C1—C2—H2A	109.1	C15—C16—H16	120.5
C1—C2—H2B	109.1	C15—C16—C17	119.08 (15)
C1—C2—C3	112.48 (12)	C17—C16—H16	120.5
H2A—C2—H2B	107.8	C12—C17—H17	120.2
C3—C2—H2A	109.1	C16—C17—C12	119.56 (14)
C3—C2—H2B	109.1	C16—C17—H17	120.2
C2—C3—H3A	109.5	C19—C18—N1	119.04 (13)
C2—C3—H3B	109.5	C19—C18—C23	120.78 (13)
H3A—C3—H3B	108.0	C23—C18—N1	120.14 (13)
C4—C3—C2	110.92 (12)	C18—C19—H19	120.0
C4—C3—H3A	109.5	C18—C19—C20	120.00 (14)
C4—C3—H3B	109.5	C20—C19—H19	120.0
C3—C4—H4A	109.4	C19—C20—H20	120.7
C3—C4—H4B	109.4	C21—C20—C19	118.67 (14)
H4A—C4—H4B	108.0	C21—C20—H20	120.7
C5—C4—C3	111.31 (12)	C20—C21—Cl1	119.45 (12)
C5—C4—H4A	109.4	C20—C21—C22	121.77 (14)
C5—C4—H4B	109.4	C22—C21—Cl1	118.74 (12)
N1—C5—C4	116.74 (12)	C21—C22—H22	120.4
C6—C5—N1	120.68 (13)	C21—C22—C23	119.24 (14)
C6—C5—C4	122.51 (13)	C23—C22—H22	120.4
C1—C6—C7	117.14 (12)	C18—C23—C22	119.46 (14)
C5—C6—C1	120.51 (13)	C18—C23—H23	120.3
C5—C6—C7	122.33 (13)	C22—C23—H23	120.3
C6—C7—H7	108.8	C25—C24—C7	119.70 (13)
C6—C7—C8	109.55 (11)	C29—C24—C7	121.73 (13)
C6—C7—C24	110.49 (12)	C29—C24—C25	118.56 (14)
C8—C7—H7	108.8	C24—C25—H25	119.5
C8—C7—C24	110.44 (12)	C26—C25—C24	120.93 (14)
C24—C7—H7	108.8	C26—C25—H25	119.5
C9—C8—C7	121.50 (13)	C25—C26—H26	119.9
C9—C8—C11	114.95 (13)	C25—C26—C27	120.17 (14)
C11—C8—C7	123.21 (12)	C27—C26—H26	119.9
N2—C9—N1	115.39 (12)	C26—C27—H27	120.3
N2—C9—C8	124.53 (13)	C28—C27—C26	119.48 (14)
C8—C9—N1	120.07 (13)	C28—C27—H27	120.3
N2—C10—N3	128.20 (14)	C27—C28—H28	119.9
N2—C10—H10	115.9	C27—C28—C29	120.14 (14)
N3—C10—H10	115.9	C29—C28—H28	119.9
N3—C11—N4	117.64 (13)	C24—C29—H29	119.6
N3—C11—C8	121.96 (13)	C28—C29—C24	120.71 (14)
N4—C11—C8	120.36 (13)	C28—C29—H29	119.6
C13—C12—N4	117.24 (13)		
			
Cl1—C21—C22—C23	174.98 (12)	C9—N1—C18—C19	99.10 (17)
F1—C15—C16—C17	−178.45 (14)	C9—N1—C18—C23	−83.32 (18)
O1—C1—C2—C3	−158.02 (14)	C9—N2—C10—N3	−2.2 (2)
O1—C1—C6—C5	−172.82 (14)	C9—C8—C11—N3	−3.7 (2)
O1—C1—C6—C7	5.6 (2)	C9—C8—C11—N4	178.79 (13)
N1—C5—C6—C1	171.52 (13)	C10—N2—C9—N1	−179.02 (13)
N1—C5—C6—C7	−6.8 (2)	C10—N2—C9—C8	0.5 (2)
N1—C18—C19—C20	174.82 (14)	C10—N3—C11—N4	179.98 (13)
N1—C18—C23—C22	−175.75 (13)	C10—N3—C11—C8	2.4 (2)
N4—C12—C13—C14	−176.98 (14)	C11—N3—C10—N2	0.7 (2)
N4—C12—C17—C16	178.02 (13)	C11—N4—C12—C13	−137.19 (15)
C1—C2—C3—C4	−51.88 (17)	C11—N4—C12—C17	44.2 (2)
C1—C6—C7—C8	−155.73 (12)	C11—C8—C9—N1	−178.33 (13)
C1—C6—C7—C24	82.38 (15)	C11—C8—C9—N2	2.2 (2)
C2—C1—C6—C5	5.6 (2)	C12—N4—C11—N3	−10.0 (2)
C2—C1—C6—C7	−175.96 (12)	C12—N4—C11—C8	167.69 (14)
C2—C3—C4—C5	51.70 (17)	C12—C13—C14—C15	−1.1 (2)
C3—C4—C5—N1	159.04 (13)	C13—C12—C17—C16	−0.6 (2)
C3—C4—C5—C6	−24.0 (2)	C13—C14—C15—F1	179.54 (14)
C4—C5—C6—C1	−5.4 (2)	C13—C14—C15—C16	−0.6 (3)
C4—C5—C6—C7	176.28 (13)	C14—C15—C16—C17	1.7 (3)
C5—N1—C9—N2	−169.69 (13)	C15—C16—C17—C12	−1.0 (2)
C5—N1—C9—C8	10.8 (2)	C17—C12—C13—C14	1.7 (2)
C5—N1—C18—C19	−81.97 (18)	C18—N1—C5—C4	−13.3 (2)
C5—N1—C18—C23	95.60 (17)	C18—N1—C5—C6	169.60 (13)
C5—C6—C7—C8	22.68 (19)	C18—N1—C9—N2	9.24 (19)
C5—C6—C7—C24	−99.22 (16)	C18—N1—C9—C8	−170.32 (13)
C6—C1—C2—C3	23.54 (19)	C18—C19—C20—C21	1.0 (2)
C6—C7—C8—C9	−23.13 (18)	C19—C18—C23—C22	1.8 (2)
C6—C7—C8—C11	163.86 (13)	C19—C20—C21—Cl1	−175.91 (12)
C6—C7—C24—C25	62.20 (17)	C19—C20—C21—C22	1.7 (2)
C6—C7—C24—C29	−116.82 (15)	C20—C21—C22—C23	−2.7 (2)
C7—C8—C9—N1	8.1 (2)	C21—C22—C23—C18	0.9 (2)
C7—C8—C9—N2	−171.39 (13)	C23—C18—C19—C20	−2.7 (2)
C7—C8—C11—N3	169.77 (13)	C24—C7—C8—C9	98.79 (15)
C7—C8—C11—N4	−7.8 (2)	C24—C7—C8—C11	−74.21 (17)
C7—C24—C25—C26	−178.69 (15)	C24—C25—C26—C27	−0.1 (3)
C7—C24—C29—C28	178.86 (14)	C25—C24—C29—C28	−0.2 (2)
C8—C7—C24—C25	−59.17 (17)	C25—C26—C27—C28	−0.2 (3)
C8—C7—C24—C29	121.81 (14)	C26—C27—C28—C29	0.4 (2)
C9—N1—C5—C4	165.57 (13)	C27—C28—C29—C24	−0.2 (2)
C9—N1—C5—C6	−11.5 (2)	C29—C24—C25—C26	0.3 (2)

### 10-(4-Chlorophenyl)-4-[(4-fluorophenyl)amino]-5-phenyl-5,8,9,10-tetrahydropyrimido[4,5-b]quinolin-6(7H)-one Hydrogen-bond geometry (Å, º)

*Cg*1 and *Cg*2 are the centroids of the C24–C29 and C18–C23 rings, respectively.

**Table:**

*D*—H···*A*	*D*—H	H···*A*	*D*···*A*	*D*—H···*A*
N4—H4···O1^i^	0.88	2.17	3.0036 (16)	159
C29—H29···O1^i^	0.95	2.46	3.3169 (18)	150
C10—H10···*Cg*1^ii^	0.95	2.74	3.5719 (18)	147
C15—F1···*Cg*2^iii^	1.36 (1)	3.16 (1)	4.4286 (18)	155 (1)

Symmetry codes: (i) −*x*, −*y*, −*z*+1; (ii) *x*+1, *y*, *z*; (iii) *x*, *y*−1, *z*−1.
